# A Systematic Review of Thermosensation and Thermoregulation in Anxiety Disorders

**DOI:** 10.3389/fphys.2021.784943

**Published:** 2021-12-06

**Authors:** Susanne Fischer, Florence Haas, Jana Strahler

**Affiliations:** ^1^Clinical Psychology and Psychotherapy, Institute of Psychology, University of Zurich, Zurich, Switzerland; ^2^Sports Psychology, Institute of Sport and Sport Sciences, Albert-Ludwigs-Universität Freiburg, Freiburg, Germany

**Keywords:** anxiety, panic, skin conductance, sweat, temperature, phobia

## Abstract

**Objectives:** Sweating, hot flushes, and blushing are symptoms frequently reported by individuals with anxiety disorders. They represent important reinforcers of anxiogenic cognitions and behaviours. One system that may be involved in the manifestation of these symptoms is the thermosensory/thermoregulatory system. The aim of the present study was to investigate to what extent individuals with anxiety disorders are characterised by alterations in this system.

**Methods:** PubMed and PsycINFO were systematically searched. Studies were eligible if they (i) assessed individuals with anxiety disorders, (ii) thermosensation or thermoregulatory effectors/outcomes, and (iii) used a case-control design.

**Results:** *N* = 86 studies were identified. There was no evidence of altered thermosensation in individuals with anxiety disorders. Regarding thermoregulatory effectors, individuals with social anxiety disorder exhibited altered cutaneous vasodilation upon pharmacological challenge; individuals with specific phobia showed increased sweating upon confrontation with phobic stimuli; individuals with panic disorder showed increased daily sweating as well as increased sweating in response to non-phobic and phobic stimuli. Regarding thermoregulatory outcomes, there was evidence for altered skin temperature in all subtypes of anxiety.

**Conclusion:** Whereas there was no evidence of altered thermoregulation in specific phobia, a subgroup of individuals with social anxiety and panic disorder appears to exhibit altered vasodilation and sweating, respectively. Longitudinal research is warranted to investigate whether this represents a vulnerability to anxiety/panic.

## Graphical Abstract

This systematic review sought to summarise studies comparing thermosensory and thermoregulatory functioning between individuals with anxiety disorders and healthy controls. Its main findings are summarised on the right side.

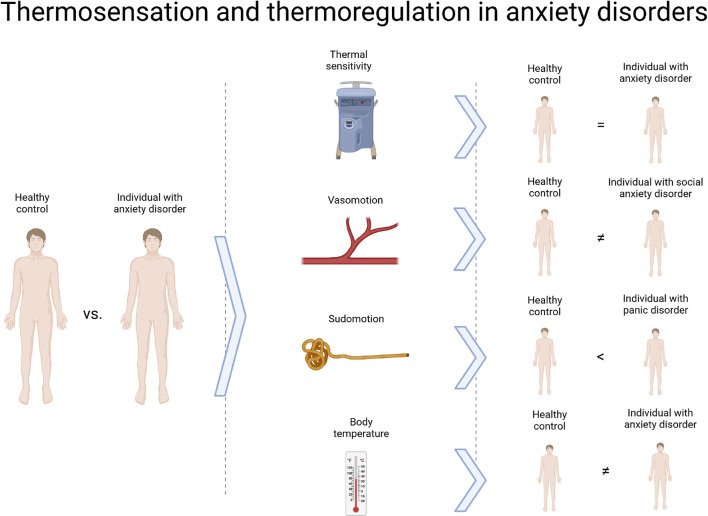

## Introduction

An estimated 7.3% of the global population are affected by anxiety disorders (Baxter et al., 2013) and these disorders constitute the second-strongest contributor to the global burden of disease (Whiteford et al., 2013). Anxiety disorders cause tremendous suffering in affected individuals and lead to frequent healthcare use and reduced productivity, thus also exerting a substantial financial burden on society (Konnopka et al., 2009).

Individuals with anxiety disorders are characterised by a wide range of *psychological* symptoms, including intense fear and/or anxiety and avoidance behaviour (APA, 2013). In addition, they report various *physical* symptoms. There is ample evidence that these individuals suffer from a number of physiological abnormalities, which may render them more vulnerable to the experience of such symptoms. For instance, reduced heart rate variability at rest is a frequent finding in individuals with anxiety disorders (see Chalmers et al., 2014 for a meta-analysis) and may increase their likelihood of experiencing cardio-respiratory symptoms and related adverse health outcomes. By contrast, little is known about the underpinnings of other bodily symptoms that are frequently experienced by these individuals, namely increased sweating, chills, hot flushes, and blushing. This is unfortunate, since these symptoms are potentially visible to others, which renders them particularly prominent reinforcers of anxiogenic cognitions and behaviours. In line with this notion, it was found that the symptom cluster of sweating and chills/hot flushes is uniquely associated with social concerns (e.g., embarrassment) in individuals with panic disorder (Drenckhan et al., 2015). Furthermore, it has repeatedly been observed that hyperhidrosis, i.e., excessive sweating, is linked to greater illness severity and disability in individuals with social anxiety disorder, most likely due to the significant social impairment it causes (Davidson et al., 2002).

One system that may be involved in the manifestation of the sweating and chills/hot flushes cluster is the thermosensory/thermoregulatory system, which is only beginning to be understood in anxiety disorders. Thermosensation, i.e., the perception of temperature, begins with thermosensory neurons located in the skin and in mucous membranes, and follows two subsequent routes: The spinothalamic pathway (e.g., Craig et al., 2000; Craig, 2018), which serves discriminatory purposes, and the spinoparabrachial pathway (e.g., Nakamura, 2011; Morrison, 2016; Morrison and Nakamura, 2019), which governs thermoregulation. Thermoregulation involves the activation of brown adipose tissue and shivering to generate heat, cutaneous vasoconstriction and piloerection to prevent heat loss, and cutaneous vasodilation and sweating to facilitate heat loss (Nakamura, 2011; Morrison, 2016). The activation of these so called “thermoeffectors” serves the purpose of maintaining core temperature at approximately 37°C. No studies to date have examined the entire thermal system in anxiety disorders. However, in the past decades, different lines of research have investigated thermosensation, the functioning of different thermoeffectors (e.g., vasoconstriction, vasodilation, and sweating), and thermoregulatory outcomes (e.g., skin and core temperature) in these individuals.

The aim of the present study was to find out to what extent individuals with anxiety disorders are characterised by alterations in thermosensory/thermoregulatory functioning, which may be a substrate for some of the most commonly experienced symptoms in this population. To this end, a systematic review of studies comparing individuals with anxiety disorders and healthy controls regarding the thermosensation, thermoeffector functioning, and thermoregulatory outcomes was undertaken following PRISMA guidelines (Moher et al., 2009).

## Methods

### Systematic Search

The systematic review was registered with PROSPERO (CRD42020149925). The databases PubMed and PsycINFO were searched from the first available year until September 2019. The search string combined terms related to anxiety disorders (e.g., “phobia”) with terms related to thermosensation/thermoregulation (e.g., “temperature”; see Supplementary Material 1). The search relied on both key words and exploded subject headings. Studies were eligible if they (i) included an adult sample of individuals with an anxiety disorder (i.e., specific phobia, social anxiety disorder, panic disorder, agoraphobia, and generalised anxiety disorder) according to the third, fourth, or fifth edition of the Diagnostic and Statistical Manual of Mental Disorders (DSM; APA, 1987, 2000, 2013) or according to the tenth edition of the International Classification of Diseases (ICD; WHO, 1992), (ii) assessed thermosensation or thermoregulatory effectors/outcomes (e.g., vasoconstriction, vasodilation, sweating, skin/core temperature) and (iii) used a case-control study design. Only studies published in German, English, Italian, Spanish, or French were screened for eligibility. All duplicates were removed and titles and abstracts were screened to exclude studies that did not meet eligibility criteria. Next, full-text articles were inspected to select eligible studies. For studies for which the abstract or the full text was not available, and for those in which the relevant outcome was not reported, the authors were contacted. To identify additional relevant studies, the reference lists of all included articles were screened manually. The systematic search was performed by all three study investigators, with disagreements regarding the inclusion of studies discussed between SF and JS until a consensus was reached.

### Data Extraction

For each included study, information about the first author, the year of publication, sample characteristics (number of participants, age, sex), thermosensory/thermoregulatory assessment and relevant results were extracted. The data were extracted by two of the study investigators (SF and FH) and a research assistant. Risk of bias was assessed using a modified version of a rating scale that was used in previous systematic reviews and meta-analyses comparing biological markers between individuals with mental disorders and healthy controls (Tak et al., 2011; Fischer and Ehlert, 2018; Fischer et al., 2019). The scale consisted of four items in total (see Table 1). The first item referred to eligibility criteria, the second to the recruitment of controls, the third to the quality of thermosensation or thermoregulation measurements, and the fourth to the handling of potential confounders used. The third item was further specified (see Supplementary Material 2). Each item was rated on a 3-point scale from 0 to 2. Therefore, the maximum attainable score was 8. The study quality was rated by two of the study investigators (FH and SF) and a research assistant. All disagreements regarding the risk of bias assessments were discussed between SF and FH until a consensus was reached.

**TABLE 1:** Quality rating scale to assess risk of bias in studies investigating thermosensory and thermoregulatory functioning in anxiety disorders; items modified from Tak et al. (2011), Fischer and Ehlert (2018), and Fischer et al. (2019).

(1) What eligibility criteria were used?	No comorbidity with major somatic diseases, no comorbidity with major mental disorders, no current medication use, 3 used (2) No comorbidity with major somatic diseases, no comorbidity with major mental disorders, no current medication use, 1–2 used (1) No comorbidity with major somatic diseases, no comorbidity with major mental disorders, no current medication use, 0 used or not clearly stated (0)
(2) How were controls recruited?	From the same population as patients (2) From a selected population, such as hospital staff or students, or not clearly stated (0)
(3) How were measures of thermosensation or thermoregulation taken?	*Thermosensation:* Measure adequate regarding equipment and setting (2) Measure adequate regarding equipment or setting (1) Measure inadequate regarding equipment and setting or not clearly stated (0)
	*Skin conductance:* Measure adequate regarding equipment, setting, time of day, accommodation period, artefact control, 4–5 fulfilled (2) Measure adequate regarding equipment, setting, time of day, accommodation period, artefact control, 2–3 fulfilled (1) Measure adequate regarding equipment, setting, time of day, accommodation period, artefact control, 0–1 fulfilled or not clearly stated (0)
	*Body temperature:* Measure adequate regarding equipment, setting, time of day, accommodation period, 3–4 fulfilled (2) Measure adequate regarding equipment, setting, time of day, accommodation period, 1–2 fulfilled (1) Measure adequate regarding equipment, setting, time of day, accommodation period, 0 fulfilled or not clearly stated (0)
(4) What confounders were used to adjust the statistical analyses?^a^	Age, sex, body mass index, caffeine, depression, 4–5 stated (2) Age, sex, body mass index, caffeine, depression, 2–3 stated (1) Age, sex, body mass index, caffeine, depression, 0–1 or not clearly stated (0)

*^a^In the case of matched groups, the variable being an exclusion criterion, or in the case of no significant impact on statistical analyses, consider confounder as adjusted.*

## Results

### Search Results

**FIGURE 1:**
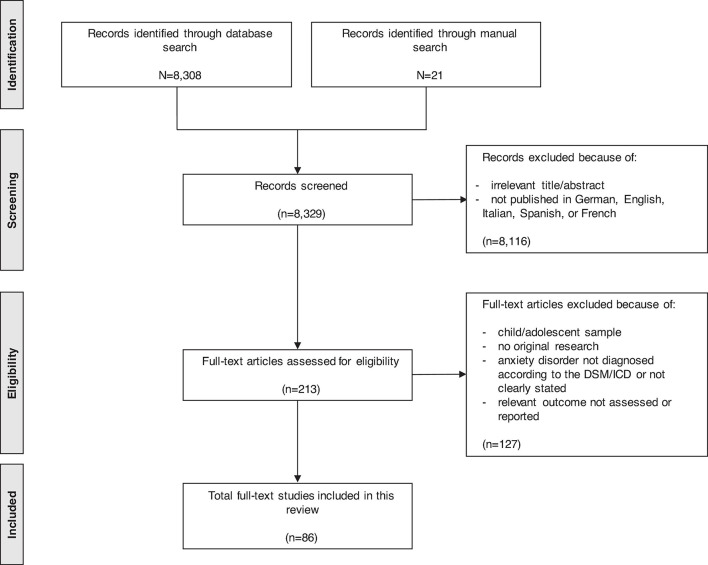
Search process.

The search process is depicted in Figure 1. The literature search yielded 8,329 records, of which 8,116 were considered irrelevant after screening of the titles and abstracts. Thus, 213 full texts were checked for eligibility, and 127 were excluded for the following reasons: child/adolescent sample, not original research, anxiety disorder not diagnosed according to the DSM/ICD as stated above, or relevant outcome not assessed/reported. Finally, a total of 86 studies were included in the systematic review. The included studies were published between 1984 and 2017. The sample sizes ranged from 12 to 236. Around two thirds of the studies included individuals with *panic disorder* (62%), one fifth included individuals with *specific phobia* (21%), one fifth included individuals with *social anxiety disorder* (20%), and one eighth included individuals with *generalised anxiety disorder* (16%).

### Thermosensation

Four studies investigated thermal sensitivity, mostly by means of thermodes and the cold pressor test. Individuals with different forms of anxiety disorders were not found to differ from healthy controls in their detection of warm or cold stimuli (Defrin et al., 2008), or in their heat (Defrin et al., 2008) or cold pain thresholds (Mostoufi et al., 2014). The null findings relating to heat and cold pain were confirmed by two studies focusing on individuals with *panic disorder* (Lautenbacher et al., 1999; Spernal et al., 2003).

### Thermoregulation

#### Vasoconstriction and Vasodilation

Two studies, both in individuals with *social anxiety disorder*, investigated cutaneous vasodilation as indicated by forearm/cheek blood flow and cheek/forehead blood flow, respectively (Katzman et al., 2003; Voncken and Bogels, 2009). These studies demonstrated that whereas socially anxious individuals’ resting forearm/cheek blood flow (Katzman et al., 2003) and social conversation and speech task cheek/forehead blood flow (Voncken and Bogels, 2009) did not differ from those of healthy controls, socially anxious individuals had comparably lower increases in forearm/cheek blood flow after a higher-dosed nicotinic challenge (Katzman et al., 2003).

#### Sweating

Seventy studies investigated sweating as indicated by skin conductance levels, non-specific skin conductance fluctuations, or skin conductance responses. Only one study used a sample of individuals with different forms of anxiety disorder (Marin et al., 2017). This study demonstrated blunted skin conductance responses during fear conditioning in individuals with anxiety disorders when compared to healthy controls.

Fifteen studies focused on patients with *specific phobia* (see Supplementary Material 3). Six studied animal phobias (40%), five situational phobias (33%), two blood-injection-injury phobias (13%), and two studied different types of phobia (13%). The risk of bias in these studies was moderate, with an average of 3 out of 8 points on the quality rating scale. The majority of studies assessing skin conductance at rest failed to find a significant difference between individuals with specific phobias and controls. Similarly, there was no evidence that individuals with specific phobias and controls differed in skin conductance when exposed to stressors unrelated to their phobia (e.g., physical exercise, cognitive tasks, aversive imagery). By contrast, studies exposing participants to phobic stimuli (in sensu or *in vivo*) found elevated skin conductance in individuals with specific phobias as compared to controls. Notably, this did not apply to studies on blood-injection-injury phobia, which did not detect any differences between individuals with specific phobias and controls. Furthermore, most studies using virtual reality scenarios did not reveal differences in skin conductance between individuals with specific phobias and controls, although those that did find differences had comparably higher quality ratings.

Thirteen studies examined patients with *social anxiety disorder* (see Supplementary Material 4), only two of which were confined to patients with generalised social anxiety disorder. Generally, the risk of bias was moderate, with studies scoring 2.7 points on average on the quality rating scale. None of the studies investigating skin conductance at rest detected any differences between patients and healthy controls. Furthermore, neither studies employing general laboratory stressors nor studies utilising social conversation or speech tasks found any differences in the skin conductance of individuals with social anxiety disorder and controls. Half of the studies employing social imagery tasks found higher skin conductance in individuals with social anxiety disorder vs. controls, whereas the other half failed to find such differences. On average, the studies yielding positive findings had a higher quality rating than those yielding null findings.

Thirty-eight studies investigated *panic disorder and/or agoraphobia* (see Supplementary Material 5) and all but one of these studies included patients with panic disorder. The risk of bias in the panic disorder/agoraphobia studies was moderate, with an average of 3 points on the quality rating scale. The majority of studies measuring resting skin conductance did not find any differences between individuals with panic disorder and healthy controls. Notably, two studies used ambulatory assessments, with one finding higher daily skin conductance levels in individuals with panic disorder as compared to controls (Doberenz et al., 2010) and the other reporting no differences in instances where stress or panic was reported (Hoehn-Saric et al., 2004). Regarding general laboratory stressors, the majority of studies exposing participants to stressful films or to social speech tasks found comparably elevated skin conductance in individuals with panic disorder, whereas the majority of studies that used cognitive tasks or negative imagery failed to find such differences. Findings regarding acoustic stimulation were equivocal, although the studies yielding positive findings had higher quality ratings on average than those yielding null findings. Regarding potentially panic-evoking physiological stimuli, different infusions (i.e., with sodium lactate and isoproterenol) as well as CO_2_ inhalation, but not hyperventilation, were accompanied by increased skin conductance. Regarding potentially panic-evoking psychological stimuli, relaxation tasks did not provoke greater increases in individuals with panic disorder’s skin conductance when compared to controls, nor did imagery exercises. By contrast, videos of panic-related scenes were linked to comparably higher skin conductance in individuals with panic disorder.

Finally, ten individual studies investigated patients with *generalised anxiety disorder* (see Supplementary Material 6). The risk of bias in the generalised anxiety disorder studies was moderate, with an average of 3.4 points on the quality rating scale. The majority of studies did not provide any evidence of altered skin conductance at baseline. This included the one study to have used ecological momentary assessment (Hoehn-Saric et al., 2004). Findings during different laboratory stressors were likewise negative, although there was some indication that individuals with generalised anxiety disorder had lower skin conductance during gambling tasks.

#### Body Temperature

Nineteen studies looked at body temperature. In a sample of individuals with various anxiety disorders, no differences in finger temperature emerged between individuals with anxiety disorders and controls at baseline and during acoustic stimulation with 60 dB (Jensen et al., 1996).

In studies focusing on *specific phobia*, it was found that individuals with flight phobia had lower finger temperature than did controls during *in vivo* flying (Wilhelm and Roth, 1998), but not during virtual flying (Wiederhold et al., 2002). The same measure did not distinguish between individuals with driving phobia and healthy controls at baseline, but individuals with driving phobia exhibited greater temperature increases between driving to the exposure situation and driving back (Alpers et al., 2005).

In studies on *social anxiety disorder*, hand, face, and/or neck temperature were found to be unaltered in the resting state (Bouwer and Stein, 1998; Edelmann and Baker, 2002), during different laboratory stress tasks (Edelmann and Baker, 2002), social conversation/speech (Edelmann and Baker, 2002; Voncken and Bogels, 2009), or social imagery tasks (Edelmann and Baker, 2002), but elevated after a nicotinic challenge (Bouwer and Stein, 1998).

Twelve studies investigated *panic disorder and/or agoraphobia* (see Supplementary Material 7). The risk of bias in these studies was moderate, with an average of 3.3 points on the quality rating scale. Neither resting skin temperature nor skin temperature responses to general laboratory stressors (e.g., physical exercise, cognitive tasks, aversive imagery) distinguished individuals with panic disorder from controls. Regarding potentially panic-evoking physiological stimuli, hyperventilation was not associated with altered skin temperature, but different infusions were linked to comparably lower skin temperature in individuals with panic disorder (Freedman et al., 1984). Regarding potentially panic-evoking psychological stimuli, most studies found that relaxation tasks were not paralleled by altered skin temperature, although one study found that individuals with panic disorder had higher skin temperatures during acoustic feedback about physiological arousal (Craske and Freed, 1995). There were no differences regarding the resting oral temperature (an indicator of core body temperature) of individuals with panic disorder and controls. Pharmacological challenge studies were equivocal, with one study reporting less pronounced decreases in oral temperature in individuals with panic disorder (Lesch, 1991; Lesch et al., 1992) and another study (with a slightly higher quality rating) reporting a null finding (Broocks et al., 2000).

## Discussion

This systematic review yielded three main findings. First, individuals with anxiety disorders were not characterised by altered thermosensation. Second, they exhibited a number of alterations in thermoeffector functioning: Individuals with social anxiety disorder exhibited altered cutaneous vasodilatory responses upon pharmacological challenge; individuals with specific phobias or social anxiety disorder showed more pronounced sweating upon confrontation with phobic stimuli; individuals with panic disorder were characterised by more intense daily sweating as well as enhanced sweating during stressful laboratory tasks and upon exposure to potentially panic-evoking stimuli. Third, there was evidence for altered skin temperature in all of these three subtypes of anxiety disorder, although, notably, nearly all studies relied on finger temperature, which is not a representative marker of skin temperature.

The first finding is of no evidence for altered thermosensation in individuals with anxiety disorders. This finding is matched by findings of equal thermal discomfort between anxious individuals and healthy controls (Defrin et al., 2008; Mostoufi et al., 2014). However, the literature is sparse and mainly consisting of studies employing thermal stimuli at noxious levels. Thermosensory neurons include both nociceptive neurons and specific thermoceptive neurons. Whereas the former respond to cold and heat at noxious levels, the latter transmit stimuli at innocuous levels (i.e., from approximately 15 to 45°C; Raison et al., 2015). The single study administering innocuous thermal stimuli and comparing responses between anxious and healthy participants yielded null findings (Defrin et al., 2008). However, this study was designed to investigate pain rather than temperature perception and may thus have been underpowered to detect subtle differences between patients with anxiety disorders and controls. Further experimental studies employing cold/warm cues at both levels of intensity and, ideally, by means of a thermode, are therefore warranted in order to provide a comprehensive account of thermosensory signalling in anxiety disorders.

The second and third findings are of altered vasodilation and sweating in subgroups of patients with anxiety disorders. Individuals with social anxiety disorder showed elevated hand, cheek and neck temperature after a topical nicotinic challenge and an attenuated forearm/cheek blood flow after a systemic nicotinic challenge. These discrepant findings point to an intricate role of vasodilation in social anxiety, depending on whether changes are triggered by peripherally or centrally acting substances. However, this finding is based on only two studies and further research examining vasoconstriction and vasodilation in situations with greater ecological validity (i.e., everyday life social interactions) is warranted to shed light on the extent to which this thermoeffector is involved in the experience of chills, hot flushes, or blushing. It is conceivable that alterations in vasoconstriction or vasodilation are exclusively linked to social anxiety in a subgroup of individuals with high levels of blushing and/or hot flushes.

In individuals with specific phobia, elevated sweating was closely linked to the presence of phobic stimuli. This suggests that, in this subtype of anxiety disorder, enhanced sweating is an epiphenomenon of intense fear. Contrary to this specific activation pattern, individuals with panic disorder showed hyper-activity in panic-provoking, in generally stressful, and in non-stressful everyday life circumstances. This finding points to a predisposition to enhanced sweating, which may provide a breeding ground for the occurrence of panic attacks. This notion is in line with evidence that panic attacks are more frequent under specific meteorological conditions (e.g., hot winds; Bulbena et al., 2005). However, at least two caveats need to be issued against this interpretation. First, none of the included studies was longitudinal, hence rendering it impossible to infer a temporal order whereby an increased propensity to sweat precedes the development of panic disorder. Second, all identified studies measured sweating by means of palmar skin conductance. This is noteworthy given that the distribution and function of sweat glands varies across the human body, with the palms constituting a particularly relevant site for emotional (but not for thermoregulatory) sweating (Boucsein et al., 2012). Furthermore, skin conductance is not solely dependent on sweat gland activity, but also on epidermal membrane properties. Further research drawing on more widespread and direct measures of sweat gland activity (e.g., by means of ventilated capsules), on behavioural thermoregulation measures (e.g., water requirements), and approaches combining resting measurements outside the laboratory with thermal challenges in the laboratory (e.g., ambient temperature manipulations) will be necessary to illuminate the extent of altered thermoregulation in panic disorder. Notably, there was little evidence for altered core temperature in panic disorder. However, the dearth of studies in this area does not allow one to exclude the possibility that more continuous long-term monitoring (e.g., over the course of 24 h) would reveal subtle differences between patients and controls. Importantly, given the respiratory abnormalities that are sometimes observed in panic disorder (Grassi et al., 2013), oral temperature may not be an ideal proxy of core temperature in this population since its reliability may be compromised by altered breathing.

The present study is the first review of the current state of research on thermal functioning in anxiety disorders. Our search was systematic and yielded a significant number of studies that provide a nuanced summary of the research in this area. However, a number of limitations need to be acknowledged. First, our search yielded relatively few studies on thermosensation and on core body temperature, which points to the need for further enquiries in these neglected areas. Likewise, the thermoregulatory research was heavily skewed toward sweating, with no research on other important thermoeffectors such as brown adipose tissue activity. Second, there was substantial methodological heterogeneity across the included studies (e.g., in terms of the laboratory tasks employed), which prevented us from undertaking meta-analyses. Third, the risk of bias review revealed a number of methodological limitations pertaining to the selected studies. For instance, not all studies excluded patients with a current major depressive episode, which is problematic given that these patients show distinct thermosensory and thermoregulatory abnormalities, including reduced temperature sensitivity, sweating, and increased core body temperature (Raison et al., 2015). Similarly, not all studies excluded medication that affects the thermal system (e.g., antidepressants; Beyer et al., 2017), and not all statistical analyses were adjusted for important confounders such as age, sex, or BMI. Further case-control research may thus benefit from consulting the methodological standards outlined in this review. Fourth, although our quality assessment scale was adapted from prior research and did not lead to any major disagreements between the raters, we did not calculate an interrater reliability coefficient for this systematic review and thus cannot provide evidence for its reliability in the context of the present study.

When taken together, the present results may suggest that, in subgroups of patients with anxiety disorders, symptoms such as hot flushes, blushing, and increased sweating may be paralleled by specific alterations in vasodilation and sweating. Recent animal research suggests that at least some of these alterations may be stress-related (Oka, 2015; Kataoka et al., 2020). Micro-longitudinal research is now warranted to investigate whether they represent a vulnerability to the experience of social anxiety and/or panic attacks in individuals already exhibiting clinically relevant levels of anxiety. Moreover, epidemiological studies may shed light on the extent to which these alterations predispose individuals toward the development of social anxiety and panic disorder and how they progress over the course of these illnesses (e.g., in terms of neural/cutaneous adaptations). Interestingly, animal research has also demonstrated that disturbances in the thermal system could be related to *psychological* symptoms inherent in anxiety disorders (Lowry et al., 2009; Hale et al., 2013; Raison et al., 2015). Further, rigorous research into thermal functioning in anxiety disorders is thus necessary to elucidate the role of this system in the development and maintenance of these debilitating conditions.

## Data Availability Statement

The original contributions presented in the study are included in the article/Supplementary Material, further inquiries can be directed to the corresponding author/s.

## Author Contributions

SF conceived the study and provided the first draft of the manuscript. SF, FH, and JS conducted the systematic search. SF and FH extracted the study data. FH and JS revised it critically for important intellectual content. All authors contributed to the article and approved the submitted version.

## Conflict of Interest

The authors declare that the research was conducted in the absence of any commercial or financial relationships that could be construed as a potential conflict of interest.

## Publisher’s Note

All claims expressed in this article are solely those of the authors and do not necessarily represent those of their affiliated organizations, or those of the publisher, the editors and the reviewers. Any product that may be evaluated in this article, or claim that may be made by its manufacturer, is not guaranteed or endorsed by the publisher.
