# Supplementary material for: A Systematic Review of Thermosensation and Thermoregulation in Anxiety Disorders
Source: Front Physiol. 2021 Dec 6;12:784943. doi: 10.3389/fphys.2021.784943 (PMC8685525; doi:10.3389/fphys.2021.784943)
Supplement: Supplementary file 1 [file Data_Sheet_1.docx]

**Supplement 1** Search terms

| PubMed | PsycINFO |
| --- | --- |
| **Anxiety disorders** | **Anxiety disorders** |
| Anxiety | Anxiety |
| «Anxiety Disorders» as MeSH | «Anxiety Disorders» as DE |
| Fear | Fear |
| GAD | GAD |
| «generalized anxiety disorder» | «generalized anxiety disorder» |
| Neurotic* | Neurotic* |
| «Panic disorder» | «Panic disorder» |
| Phobi* | Phobi* |
| **Thermosensation/-regulation** | **Thermosensation/-regulation** |
| «body heat» | «body heat» |
| «Body Temperature» as MeSH | «Body Temperature» as DE |
| «brown adipose tissue» | «brown adipose tissue» |
| «cold pain» | «cold pain» |
| EDA | EDA |
| electrodermal | electrodermal |
| «galvanic skin response» | «galvanic skin response» |
| «Galvanic Skin Response» as MeSH | «Galvanic Skin Response» as DE |
| «goose bumps» | «goose bumps» |
| «goose pimples» | «goose pimples» |
| «heat pain» | «heat pain» |
| «laser-Doppler-flowmetry» | «laser-Doppler-flowmetry» |
| «peripheral blood flow» | «peripheral blood flow» |
| piloerection | piloerection |
| plethysmograph* | plethysmograph* |
| SCL | SCL |
| SCR | SCR |
| shiver* | shiver* |
| «skin blood flow» | «skin blood flow» |
| «skin conductance» | «skin conductance» |
| sweat* | sweat* |
| «sweaty hand» | «sweaty hand» |
| temperature | temperature |
| thermoreg* | thermoreg* |
| thermal | thermal |
| vasoconstrict* | vasoconstrict* |
| vasodilat* | vasodilat* |

**Supplement 2** Specification of item 3 of the quality rating scale to assess risk of bias in studies investigating thermosensory and thermoregulatory functioning in anxiety disorders

1. **Equipment** needs to be specified regarding

- sensor characteristics (i.e., type, area of contact)
- signal evaluation (i.e., sampling rate)
- recording site
- additionally for skin conductance: method of measurement

1. **Setting**

- room temperature
- room humidity
- additionally for skin conductance: disturbing stimuli (e.g., noise)
- additionally for temperature: postural variation (i.e., supine, standing, sitting)

1. **Time of day**

- Thermoregulation: standardised between groups

1. **Accommodation period**

- Thermoregulation: at least 30 minutes

1. **Artefact control**

- Skin conductance: body movement, irregular breathing, speech, electrical/system hum

**Supplement 3** Characteristics of included studies investigating skin conductance in patients with specific phobia

| Study | Patients | Controls | Measurement | Main results | Risk of bias (0-8) |
| --- | --- | --- | --- | --- | --- |
| Wessel and Merckelbach (1998) | N=22 (all female)  Mean age: 28.1 years (range: 17-56)  Inclusion: specific phobia (spiders) according to the DSM-IV | N=24 (all female)  Mean age: 31.6 years (range: 17-52) | At baseline, during the presentation of two bulletin boards (phobic stimuli in the centre vs. scattered)  Second and third finger of the non-dominant hand  Schwartzer | Patients had a higher number of non-specific skin conductance fluctuations during the presentation | 3 |
| Wilhelm and Roth (1998) | N=14 (all female)  Mean age: 38.2±9.9 years  Inclusion: simple phobia (flying) according to the DSM-III-R  Exclusion: cardiac or respiratory disease, medication affecting the cardiovascular system | N=15 (all female)  Mean age: 38.4±11.9 years | At baseline, during exercise, and during flying  Middle phalanges of digits 3 and 4 of the left hand  Vitaport | Patients had higher skin conductance levels and a higher number of non-specific skin conductance fluctuations during flying | 4 |
| Cuthbert et al. (2003) | N=28 (23 female, 5 male)  Mean age: 33.2±13.2 years  Inclusion: specific phobia according to the DSM-III-R  Exclusion: active psychotic symptoms, health problems compromising recordings | N=24 (15 female, 9 male)  Mean age: 34.2±9.7 years | At baseline, during an imagery task (neutral, physical fear, social fear, personal fear)  Hypothenar eminence of the non-dominant hand  Coulbourn | No differences in skin conductance levels between patients and controls | 3 |
| Wikstrom, Lundh, Westerlund, and Hogman (2004) | N=40 (all female)  Mean age: 33.3±10.1 years  Inclusion: specific phobia (snakes) according to the DSM-IV | N=21 (all female)  Mean age: 34±11.2 years | During a lexical decision task  Biopac | No differences in skin conductance responses between patients and controls | 1 |
| Alpers, Wilhelm, and Roth (2005) | N=21 (all female)  Mean age: 46.6 years  Inclusion: specific phobia (driving) according to the DSM-IV  Exclusion: psychotic disorder, major depression or dysthymia, cardiac, neurologic or respiratory disease, obesity, psychoactive medication, medication affecting the cardiovascular system | N=17 (all female)  Mean age: 46.3 years | At baseline, during approaching a driving test, during a driving test, and during returning from a driving test  Middle phalanges of digits of the left hand  Vitaport | Patients had higher skin conductance levels and more non-specific skin conductance fluctuations at baseline and during driving, as well as higher skin conductance levels between approaching and returning from the driving test | 5 |
| Muehlberger, Petrusek, Herrmann, and Pauli (2005) | N=20 (15 female, 5 male)  Mean age: 39.8±9.5 years  Inclusion: specific phobia (flying) according to the DSM-IV  Exclusion: pregnancy, cardiovascular disease, medication | N=22 (17 female, 5 male)  Mean age: 38.0±8.8 years | At baseline, during virtual flying  Hypothenar and thenar eminences of the non-dominant hand  Vitaport | Patients had higher skin conductance levels during virtual flying | 4 |
| Knopf and Possel (2009) | N=46 (all female)  Mean age: 34.4±9.5 years  Inclusion: specific phobia (spiders) according to the DSM-IV and scoring >90^th^ percentile of the Spider Phobia Questionnaire  Exclusion: smoking, pregnancy, shift-work, mental disorders, acute or chronic health problems, medication influencing the immune or hormone system | N=44 (all female)  Mean age: 36.8±6.9 years | During photograph presentation (neutral, positive, and phobic stimuli)  Hypothenar eminence of the non-dominant hand  Contact Precision Instruments | Patients had greater skin conductance responses to the phobic stimuli | 4 |
| Sartory, Heinen, Wannemüller, Lohrmann, and Jöhren (2009) | N=90 (54 female, 36 male)  Mean age: 35.1±9.1 years  Inclusion: specific phobia (dental) according to the DSM-IV  Exclusion: anxiolytic medication or medication influencing the cardiovascular system | N=30 (18 female, 12 male)  Mean age: 29.6±7.5 years | At baseline, during acoustic stimulus presentation (neutral and phobic stimuli)  Hypothenar and thenar eminences of the non-dominant hand  Vitaport | No differences in non-specific skin conductance fluctuations and skin conductance responses between patients and controls | 1 |
| Ritz, Wilhelm, Meuret, Gerlach, and Roth (2011) | N=12 (9 female, 3 male)  Mean age: 37.3 years (range: 21-57)  Inclusion: specific phobia (blood, injection, injury) according to the DSM-IV  Exclusion: smoking, mental disorders, abnormal electrocardiogram, acute or chronic respiratory diseases, history of epilepsy or seizures | N=14 (10 female, 4 male)  Mean age: 36.4 years (range: 22-57) | During film presentation (negative, neutral, positive, phobic)  Hypothenar and thenar eminences of the left hand  Vitaport | No differences in skin conductance levels between patients and controls | 1 |
| McTeague, Lang, Wangelin, Laplante, and Bradley (2012) | N=160 (sex distribution not stated)  Mean age: 35±14 years (single fear principal specific phobia), 36.8±11.9 years (multiple fears principal specific phobia), 36.2±12.8 years (non-principal specific phobia)  Inclusion: specific phobia according to the DSM-IV | N=76 (50 female, 26 male)  Mean age: 31.8±11.6 years | At baseline, during an imagery task (neutral, panic attack, survival threat, personal threat)  Hypothenar eminence of the non-dominant hand  Coulbourn | Patients had higher skin conductance levels during personal threat imagery | 3 |
| Peperkorn, Alpers, and Muhlberger (2014) | N=48 (37 female, 11 male)  Mean age: 26.1±8.8 years  Inclusion: specific phobia (spiders) according to the DSM-IV | N=48 (37 female, 11 male)  Mean age: not stated | At baseline, during presentation of a virtual spider  Middle phalanx of the index and middle finger  Varioport | No differences in skin conductance levels between patients and controls | 3 |
| Notzon et al. (2015) | N=41 (37 female, 4 male)  Mean age: 27.5±9.5 years  Inclusion: specific phobia (spiders) according to the DSM-IV, ≥16 points on the Spider Phobia Questionnaire  Exclusion: pregnancy, mental disorders, severe somatic diseases, intake of psychotropic medication | N=42 (37 female, 5 male)  Mean age: 25.4±7.4 years | During presentation of a virtual spider  Hypothenar and thenar eminences of the non-dominant hand  V-Amp | Patients had higher skin conductance levels during the presentation of the virtual spider | 4 |
| Diemer, Lohkamp, Muhlberger, and Zwanzger (2016) | N=40 (30 female, 10 male)  Mean age: 37.9± 13.9 years  Inclusion: specific phobia (heights) according to the DSM-IV  Exclusion: pregnancy, substance use or mood disorder, suicidal ideation, history of psychotic disorder, epilepsy or other disease of the central nervous system, migraine, history of heart disease, psychoactive drugs or psychotherapy | N=40 (28 female, 12 male)  Mean age: 33.7±11.9 years | At baseline, during a virtual height scenario  V-Amp | No differences in skin conductance levels between patients and controls | 3 |
| Li and Graham (2016) | N=34 (all female)  Mean age: 22.7±0.9 years (high oestradiol), 20.5±0.8 years (low oestradiol)  Inclusion: specific phobia (spiders) according to the DSM-IV  Exclusion: endocrinological conditions | N=26 (all female)  Mean age: 22.8±1.3 years (high oestradiol), 20.5±0.5 years (low oestradiol) | At baseline, during fear conditioning and extinction  Distal phalanx of the index and middle fingers of the non-dominant hand  ADInstruments | Patients had greater skin conductance responses during parts of the fear conditioning | 3 |
| Shiban, Peperkorn, Alpers, Pauli, and Muhlberger (2016) | N=48 (40 female, 8 male)  Mean age: 38.7±15.6 years  Inclusion: specific phobia (enclosed spaces) according to the DSM-IV  Exclusion: mental disorders | N=48 (40 female, 8 male)  Mean age: 35±15.1 years | During a virtual enclosed space scenario  Middle phalanx of the index and middle finger  Varioport | No differences in skin conductance levels between patients and controls | 3 |

DSM=Diagnostic and Statistical Manual of Mental Disorders

**Supplement 4** Characteristics of included studies investigating skin conductance in patients with social anxiety disorder

| Study | Patients | Controls | Measurement | Main results | Risk of bias (0-8) |
| --- | --- | --- | --- | --- | --- |
| Strauman (1989) | N=12 (6 female, 6 male)  Mean age: 30.7 years  Inclusion: social phobia according to the DSM-III-R  Exclusion: mental disorders. | N=15 (5 female, 10 male)  Mean age: 20.7 years | During priming with different (self-referential) attributes  Medial phalanges of the second and third fingers of the non-dominant hand  Grass or Beckman | Patients had more non-specific skin conductance fluctuations during priming with ought-discrepant attributes | 2 |
| Gerlach, Wilhelm, Gruber, and Roth (2001) and Gerlach, Wilhelm, and Roth (2003) | N=30 (17 female, 13 male)  Mean age: 40.1±12.1 years  Inclusion: social phobia according to the DSM-IV  Exclusion: medication | N=14 (7 female, 7 male)  Mean age: 39.5±10.9 years | At baseline, while watching, together with two people, a video recording of oneself singing, a conversation with a person of the opposite sex, and a talk about a minimally prepared topic  Middle phalanges of digits 3 and 4 of the left hand  Vitaport | No differences in skin conductance levels and non-specific skin conductance fluctuations between patients and controls | 3 |
| Wilhelm, Gerlach, and Roth (2001) | N=38 (24 female, 14 male)  n=24 social phobia  Mean age: total sample 36.8±11.3  Inclusion: social phobia according to the DSM-IV  Exclusion: psychoactive or cardiovascularly active medication in the 2 weeks before testing | N=24 (15 female, 9 male)  Mean age: 38.4±10.1 years | During fast breathing  Middle phalanges of digits 3 and 4 of the left hand | No differences in skin conductance levels between patients with social phobia and controls | 3 |
| Edelmann and Baker (2002) | N=36 (33 female, 3 male)  n=18 generalised social phobia  Mean age: total sample 46.9±11.6 years  Inclusion: social phobia according to the DSM-III-R  Exclusion: panic disorder, organic mental disorder, substance abuse or dependence, major depression, history of psychotic disturbances | N=18 (15 female, 3 male)  Mean age: total sample 46.9±11.6 years | At baseline, during physical exercise, a mental arithmetic task, a mental imagery task involving a personally relevant situation, and a social conversation  Two fingers of the non-dominant hand  Biopac | No differences in skin conductance levels and non-specific skin conductance fluctuations between patients with social phobia and controls | 3 |
| Hermann, Ziegler, Birbaumer, and Flor (2002) | N=14 (all male)  Mean age: 31.1±6.6 years  Inclusion: generalised social phobia according to the DSM-III-R | N=19 (all male)  Mean age: 27.2±6.4 years | During fear conditioning and extinction  Hypothenar and thenar eminences of the non-dominant hand  Coulbourn | Patients had smaller skin conductance responses during fear conditioning | 1 |
| Cuthbert et al. (2003) | N=30 (13 female, 17 male)  Mean age: 32±11.1 years  Inclusion: social phobia according to the DSM-III-R  Exclusion: active psychotic symptoms, health problems compromising recordings | N=24 (15 female, 9 male)  Mean age: 34.2±9.7 years | At baseline, during an imagery task (neutral, physical fear, social fear, personal fear)  Hypothenar eminence of the non-dominant hand  Coulbourn | Patients had greater skin conductance levels during social fear imagery | 3 |
| McTeague et al. (2009) | N=75 (49 female, 26 male)  Mean age: 32.2±13.8 years (circumscribed social phobia), 30.1±13.2 years (generalised non-depressed social phobia), 30.4±9.4 years (generalised depressed social phobia)  Inclusion criteria: social phobia according to the DSM-IV  Exclusion: psychosis or major physical diseases | N=75 controls (49 female, 26 male)  Mean age: 31.8±11.6 years | At baseline, during an imagery task (neutral, social threat, survival threat, personal fear)  Hypothenar eminence of the non-dominant hand  Coulbourn | Patients had higher skin conductance levels during social threat and personal fear imagery | 4 |
| Voncken and Bogels (2009) | N=60 (28 female, 32 male)  Mean age: 31.7±9.4 years  Inclusion: social phobia according to the DSM-IV  Exclusion: anxiolytic medication 4 days prior to testing | N=23 controls (11 female, 12 male)  Mean age: 32.5±11.3 years | During a social speech and conversation task  Middle phalanges of the middle and ring finger of the non-dominant hand  Vitaport | Patients had a lower increase in skin conductance levels in response to the social task | 1 |
| Burkhardt, Wilhelm, Meuret, Blechert, and Roth (2010) | N=19  Mean age: 39.5±10.6 years  Inclusion: social phobia according to the DSM-IV  Exclusion: lifetime history of bipolar disorder, psychosis, mental disability, drug dependence or abuse, somatic diseases that might affect the physiological measurements | N=20  Mean age: 38.4±10.9 years | During 15 min of quiet sitting  Middle phalanges of the left index and middle fingers | No differences in skin conductance levels between patients and controls | 4 |
| Moscovitch, Suvak, and Hofmann (2010) | N=39 (15 female, 24 male)  Mean age: total sample 30.6±10.3 years  Inclusion: generalised social phobia according to the DSM-IV  Exclusion: substance abuse, psychotic, manic, suicidal, homicidal, receiving psychotherapeutic treatment, change in medication status within the previous month | N=39 (15 female, 24 male)  Mean age: total sample 30.6±10.3 years | At baseline, during a social speech task  Middle phalanges of the third and fourth fingers of the left hand  James Long | No differences in skin conductance levels between patients and controls | 3 |
| Owens and Beidel (2015) | N=21 (11 female, 10 male)  Mean age: 20.9±2.2 years  Inclusion: social phobia according to the DSM-IV, Clinician Severity Rating ≥4 on the Anxiety Disorders Interview Schedule  Exclusion: alcohol or substance abuse or psychosis, lifetime bipolar disorder, suicidal ideation, unstable or serious somatic diseases, medication intake interfering with measures | N=24 (13 female, 11 male)  Mean age: 19.4±1.7 years | During an impromptu speech in front of an in vivo and virtual reality audience  MindWare Psychophysiological Ambulatory system | No differences in skin conductance levels and skin conductance responses between patients and controls | 3 |
| Sansen, Iffland, and Neuner (2015) | N=23  Mean age: 25.2±4.7 years  Inclusion: social phobia according to the DSM-IV  Exclusion: substance dependence, suicidality, lifetime psychosis | N=24  Mean age: 22.8±4.1 years | During an imagery task (standardised and personalised scripts of non-social and social situations)  Hypothenar and thenar eminences of the non-dominant hand  Varioport | No differences in skin conductance levels between patients and controls | 1 |
| Ahrens et al. (2016) | N=26 (8 female, 18 male)  Mean age: 26.5±8.4 years  Inclusion: social phobia according to the ICD-10  Exclusion: pregnancy, current use of illicit drugs, suicidal ideation, history of alcohol or substance abuse, history of psychosis or delusional disorders, somatic diseases interfering with the objectives of the study | N=29 (12 female, 17 male)  Mean age: 27.7±7.1 years | During habituation, fear acquisition, and fear generalisation  Hypothenar eminence of the non-dominant hand  V-Amp | Patients had greater skin conductance responses during habituation | 4 |

DSM=Diagnostic and Statistical Manual of Mental Disorders

**Supplement 5** Characteristics of included studies investigating skin conductance in patients with panic disorder

| Study | Patients | Controls | Measurement | Main results | Risk of bias (0-8) |
| --- | --- | --- | --- | --- | --- |
| Freedman, Ianni, Ettedgui, Pohl, and Rainey (1984) | N=8 (4 female, 4 male)  Inclusion: panic disorder according to the DSM-III | N=9 (4 female, 5 male) | During infusion (placebo, sodium lactate, isoproterenol)  Fourth and fifth finger of the right hand  Grass | Patients had higher skin conductance levels after all three infusions | 1 |
| Roth et al. (1986) | N=37 (all female)  Mean age: 41.5 (range: 21-63)  Inclusion: agoraphobia with panic attacks according to the DSM-III  Exclusion: antidepressants | N=19 (17 female, 2 male)  Mean age: 41.3 years | At baseline, during acoustic stimulation with between 75 and 105 dB  Thenar eminence of the left hand | Patients had higher skin conductance levels and more non-specific skin conductance fluctuations at baseline and higher skin conductance levels during acoustic stimulation | 3 |
| Albus, Braune, Hohn, and Scheibe (1988) | N=27 (11 female, 8 male)  Mean age: 37.4±5.7 years  Inclusion: panic disorder or agoraphobia with panic attacks according to the DSM-III  Exclusion: history of serious medical illness, drugs within 3 weeks of testing | N=10 (6 female, 4 male)  Mean age: 38.1±5.3 years | At baseline, during the presentation of two stressful videos (horror and panic attack scenes) | Patients had higher skin conductance levels and greater skin conductance responses at baseline and during stress | 1 |
| Roth, Ehlers, Taylor, Margraf, and Agras (1990) | N=38 (28 female, 10 male)  Mean age: 35.2 years  Inclusion: panic disorder or agoraphobia with panic attacks according to the DSM-III, at least one panic attack in each of the 3 weeks prior to entering the study  Exclusion: poor hearing, psychoactive drugs 2 weeks prior to testing | N=29 (22 female, 7 male)  Mean age: 35.5 years | During an acoustic stimulation with either 75 or 100 dB  Hypothenar and thenar eminences of the non-dominant hand | Patients had higher skin conductance levels, more non-specific skin conductance fluctuations, and greater skin conductance responses during acoustic stimulation | 5 |
| Argyle (1991) | N=10  Inclusion: panic disorder according to the DSM-III  Exclusion: organic illness, cyclothymia or obsessive-compulsive disorder, history of psychosis, recent history of alcohol or drug abuse, benzodiazepines | N=10  Mean age: 34.6 years | In a sound-attenuated room and in an office  Middle phalanges of the index and middle finger of the non-dominant hand  Custom-built equipment | No differences in skin conductance levels between patients and controls | 1 |
| Hoehn-Saric, McLeod, and Zimmerli (1991) | N=18 (13 female, 5 male)  Mean age: 37.5±8.9 years  Inclusion: panic disorder according to the DSM-III-R, at least one panic attack per week during the 3 weeks prior to the study  Exclusion: abnormal urine or blood values, abnormal electrocardiogram, hypertension, medication affecting the central or autonomic nervous system | N=18 (13 female, 5 male)  Mean age: 35.7±9.7 years | At baseline, during a divided attention task and during a risk-taking task  Middle phalanges of the index and middle finger of the non-dominant hand  J&J Company | No differences in skin conductance levels and non-specific skin conductance fluctuations between patients and controls | 3 |
| Beck, Stanley, Averill, Baldwin, and Deagle (1992) | N=20 (16 female, 4 male)  Mean age: 38.3±11.8 years  Inclusion: panic disorder according to the DSM-III-R  Exclusion: severe agoraphobia, enrolled in psychotherapy at the time of evaluation, intake of medication | N=20 (16 female, 4 male)  Mean age: 36.8±12.7 years | At baseline, during a verbal recognition task  Middle phalanges of the first two fingers of the left hand  Grass | Patients had higher skin conductance levels at baseline | 2 |
| Bruce, Scott, Shine, and Lader (1992) | N=12 (5 female, 7 male)  Mean age: 35 years  Inclusion: panic disorder according to the DSM-III  Exclusion: pregnancy, substance abuse, psychotropic drugs (other than anxiolytics) | N=12 (6 female, 6 male)  Mean age: 34 years | During a caffeine challenge | No differences in skin conductance levels between patients and controls | 3 |
| Passchier, Verheij, Tulen, Timmerman, and Pepplinkhuizen (1992) | N=15 (13 female, 2 male)  Mean age: 44±10.7 years  Inclusion: panic disorder according to the DSM-III  Exclusion: medication intake within 1 week (except benzodiazepines) | N=24 (12 female, 12 male)  Mean age: 31±8.4 years | At baseline, during venepuncture  Index and middle finger | No differences in skin conductance levels and non-specific skin conductance fluctuations between patients and controls | 1 |
| Roth et al. (1992) | N=52 (38 female, 14 male)  Mean age: 34.4 years  Inclusion: panic disorder or agoraphobia with panic attacks according to the DSM-III, at least one panic attack per week during the 3 weeks prior to the study  Exclusion: extreme physical conditioning, primary major depression, significant health problems, drugs in the 4 days prior to study participation | N=26 (19 female, 7 male)  Mean age: 34.5 years | At baseline, during a cold pressor test, a mental arithmetic test, and a CO_2_ inhalation  Hypothenar and thenar eminences of the non-dominant hand | Patients had more non-specific skin conductance fluctuations before the CO_2_ inhalation | 5 |
| Borden, Lowenbraun, Wolff, and Jones (1993) | N=19 (10 female, 9 male)  Mean age: 43.3 years (range: 31-64)  Inclusion: panic disorder according to the DSM-III  Exclusion: mental disorders | N=20 (10 female, 10 male)  Mean age: 20.3±4.11 years | At baseline, during relaxation, and during a mental arithmetic task  Second and third finger of the non-dominant hand  J&J Company | Patients had lower skin conductance levels at baseline, during relaxation, and during mental arithmetic | 3 |
| Braune, Albus, Frohler, Hohn, and Scheibe (1994) | N=27 (16 female, 11 male)  Mean age: 37.7±7.3 years  Inclusion: panic disorder according to the DSM-III-R  Exclusion: psychoactive medication within 3 weeks of entering the study | N=10 (6 female, 4 male)  Mean age: 37.1 years (range: 23-50) | During anticipatory stress  Palm of the left hand | Patients had higher skin conductance levels and greater skin conductance responses during anticipatory stress | 0 |
| Whittal and Goetsch (1995) | N=13 (all female)  Mean age: 32.6±12.0 years  Inclusion: panic disorder according to the DSM-III  Exclusion: alcohol abuse and dependence | N=16  Mean age: 20.1±0.7 years | At baseline, during hyperventilation  Non-dominant hand  Grass | No differences in skin conductance levels and skin conductance responses between patients and controls | 2 |
| Hoehn, Braune, Scheibe, and Albus (1997) | N=33 (19 female, 14 male)  Mean age: 38.1±7.1 years  Inclusion: panic disorder according to the DSM-III-R  Exclusion: medication within 3 weeks prior to the study | N=10 (5 female, 5 male)  Mean age: 37.1±8.1 years | At baseline, during four different stressors (frightening video scenes, mental arithmetic task, panic attack video scene, preparation and delivery of a free speech)  Hypothenar and thenar eminences of the left hand | Patients had higher skin conductance levels at baseline and during all four stress tasks | 3 |
| Roth, Wilhelm, and Trabert (1998a) | N=14 (8 female, 6 male)  Mean age: 41.6±10.2 years  Inclusion: panic disorder according to the DSM-IV  Exclusion: psychoactive or cardiovascularly active medication in the 2 weeks prior to testing | N=15 (9 female, 6 male)  Mean age: 40.3±12.6 years | During talking, relaxation, and talking  Middle phalanges of digits 3 and 4 of the left hand  Vitaport | Patients had less pronounced decreases in skin conductance levels during the relaxation task | 4 |
| Roth, Wilhelm, and Trabert (1998b) | N=19  Mean age: 43.5±9.6 years  Inclusion: panic disorder according to the DSM-III-R  Exclusion: depressive episode, respiratory diseases, psychoactive or cardiovascularly active medication in the 2 weeks before testing | N=22  Mean age: 44.8±16.4 years | At baseline, during breath holding  Vitaport | No differences in skin conductance levels between patients and controls | 4 |
| Bystritsky, Craske, Maidenberg, Vapnik, and Shapiro (2000) | N=35 (18 female, 17 male)  Mean age: 35.8±10 years  Inclusion: panic disorder according to the DSM-III-R  Exclusion: pregnancy, organic brain damage, alcohol or drug abuse within the past 6 months, psychosis, major depressive episode, personality disorder, significant health impairments | N=24 (12 female, 12 male)  Mean age: 34.6±10 years | At baseline, during CO_2_ inhalation  First two fingers of the left hand  Beckman | Patients had higher skin conductance levels at baseline and greater skin conductance responses during CO_2_ inhalation | 5 |
| Bystritsky, Maidenberg, Craske, Vapnik, and Shapiro (2000) | N=48 (28 female, 20 male)  Mean age: 35.8±10.6 years  Inclusion: panic disorder according to the DSM-III-R  Exclusion: pregnancy, organic brain damage, alcohol or drug abuse, psychosis, current major depressive episode, personality disorder,  significant health impairments, use of psychotropic drugs | N=24 (13 female, 11 male)  Mean age: 34.5±10.7 years | At baseline, during various tasks (relaxation, orthostatic challenge, hyperventilation, Valsalva, acoustic stimulation, mental arithmetic, handgrip, knee-bends, imagery)  Middle and fourth finger of the right hand  J&J Company | No differences in skin conductance levels between patients and controls | 4 |
| Sigmon et al. (2000) | N=24 (all female)  Mean age: 29.6 years  Inclusion: panic disorder without agoraphobia according to the DSM-lIl-R | N=18 (all female)  Mean age: 28 years | At baseline, during imagination (neutral and anxiety-provoking scenes)  Two middle fingers of the non-dominant hand  Contact Precision Instruments | No differences in baseline skin conductance levels between patients and controls | 3 |
| Craske, Lang, Tsao, Mystkowski, and Rowe (2001) | N=90  Mean age: total sample 34.7±9.5 years  Inclusion: panic disorder according to the DSM-IV  Exclusion: pregnancy, severe obesity, hearing impairment, substance abuse, psychotic disorder, bipolar disorder, dissociative states, history of post-traumatic stress disorder, neurological, cardiovascular, respiratory or renal diseases, pheochromocytoma, hypo- or hyperthyroidism, amphetamine intoxication, psychoactive medication | N=16  Mean age: total sample 34.7±9.5 years | At baseline, during a cardiac tracking, a meditative relaxation task, and a hyperventilation task  Middle phalanges of the second and third finger of the non-dominant hand  Coulbourn | No differences in skin conductance levels between patients and controls | 4 |
| Del-Ben et al. (2001) | N=15 (8 female, 7 male)  Mean age: 28.8±8.4 years  Inclusion: panic disorder according to the DSM-IV  Exclusion: substance abuse, depression, other anxiety disorders, somatic diseases, current use of medication (except oral contraceptives) | N=9 (3 female, 6 male)  Mean age: 27.4±7.4 years | At baseline, during habituation, fear acquisition, and extinction  Middle phalanx of the index and middle finger of the left hand  Contact Precision Instruments | Patients had more non-specific skin conductance fluctuations during habituation | 2 |
| Wilhelm, Gerlach, et al. (2001) | N=38 (24 female, 14 male)  n=14 panic disorder  Mean age: total sample 36.8±11.3 years  Inclusion: panic disorder according to the DSM-IV  Exclusion: psychoactive or cardiovascularly active medication in the 2 weeks before testing | N=24 (15 female, 9 male)  Mean age: 38.4±10.1 years | During fast breathing  Middle phalanges of digits 3 and 4 of the left hand | Patients with panic disorder had a slower decline in skin conductance levels after the fast breathing | 3 |
| Wilhelm, Trabert, and Roth (2001a) and Wilhelm, Trabert, and Roth (2001b) | N=16 (11 female, 5 male)  Mean age: 44±9 years  Inclusion: panic disorder according to the DSM-III  Exclusion: major depressive episode, epileptic, respiratory, or cardiovascular disease, psychoactive or cardiovascularly active medication in the 2 weeks prior to testing | N=19 (12 female, 7 male)  Mean age: 43.7±16.1 years | During 30 min of quiet sitting  Digits 3 and 4 of the left hand  Vitaport | No differences in skin conductance levels or non-specific skin conductance fluctuations between patients and controls | 3 |
| Cuthbert et al. (2003) | N=26 (16 female, 10 male)  Mean age: 32±11.1 years  Inclusion: panic disorder with agoraphobia according to the DSM-III-R  Exclusion: active psychotic symptoms, health problems compromising recordings | N=24 (15 female, 9 male)  Mean age: 34.2±9.7 years | At baseline, during an imagery task (neutral, physical fear, social fear, personal fear)  Hypothenar eminence of the non-dominant hand  Coulbourn | Patients had higher skin conductance levels at baseline | 3 |
| Hoehn-Saric, McLeod, Funderburk, and Kowalski (2004) | N=26 panic disorder (22 female, 4 male)  Mean age: 36.1±8.4  N=40 generalised anxiety disorder (29 female, 11 male)  Mean age: 39.6±9.2  Inclusion: panic disorder or generalised anxiety disorder according to the DSM-IV, a score ≥38 on the trait version of the State Trait Anxiety Inventory and ≥18 on the Hamilton Rating Scale for Anxiety, and at least 1 panic attack per week in the 4 weeks prior to testing (patients with panic disorder only)  Exclusion: mental disorders (except mild phobias), medication affecting the central and autonomic nervous system in the 2 weeks prior to testing | N=24 (17 female, 7 male)  Mean age: 31.6±7.7 | Ambulatory monitoring between 9 am and 5 pm  2 fingers of the non-dominant hand | No differences in skin conductance levels between patients and controls when stress or panic was reported | 5 |
| Parente, Garcia-Leal, Del-Ben, Guimaraes, and Graeff (2005) | N=30 (15 female, 15 male)  Mean age: 31.2±2.2 years  Inclusion: panic disorder according to the DSM-IV  Exclusion: pregnancy, somatic disease, medication (except antidepressants) | N=16 (7 female, 9 male)  Mean age: 34.5±3.2 years | At baseline, during the preparation and performance of speech about an emotionally neutral topic  Contact Precision Instruments | No differences in non-specific skin conductance fluctuations between patients and controls | 3 |
| Blechert, Michael, Grossman, Lajtman, and Wilhelm (2007) | N=26  Mean age: 39.4±10.7 years  Inclusion: panic disorder according to the DSM-IV  Exclusion: drug abuse or dependence, history of psychosis, bipolar disorder, history of conditions that might affect the physiological systems under study, use of medication with strong autonomic effects | N=32 controls  Mean age: 42.1±8.5 years | At baseline, during threat of shock  Middle phalanges of the index and middle finger of the left hand  Biopac | No differences in skin conductance between patients and controls | 4 |
| Michael, Blechert, Vriends, Margraf, and Wilhelm (2007) | N=39  Mean age: 40.3±10.6 years  Inclusion: panic disorder according to the DSM-IV  Exclusion: alcohol or other substance abuse in the past year, psychotic disorders, bipolar disorder, history of conditions that might affect the physiological systems under study, use of medication with strong autonomic effects | N=33  Mean age: 42.6±8.96 years | At baseline, during habituation, fear acquisition, and extinction  Middle phalanges of the index and middle fingers of the left hand  Biopac | Patients had a lower decrease in skin conductance responses between acquisition and extinction | 4 |
| Wollburg, Meuret, Conrad, Roth, and Kim (2008) | N=35  Mean age: 38.7±12.3 years  Inclusion: panic disorder according to the DSM-IV | N=33  Mean age: 39.6±11.2 years | At baseline, during hyperventilation (20 mmHg vs. 25 mmHg)  Middle phalanges of the index and middle finger of the non-dominant hand | No differences in skin conductance levels and non-specific skin conductance fluctuations between patients and controls | 3 |
| Lopes et al. (2009) | N=31 (23 female, 8 male)  Mean age: 34.0 ± 10.4 years  Inclusion: panic disorder according to the DSM-IV  Exclusion: comorbid mental disorder or clinical condition that could influence the results, psychotropic medication in the 2 weeks prior to testing, fluoxetine in the 5 weeks prior to testing | N=29 (18 female, 11 male)  Mean age: 30.1 ± 11.0 years | During the presentation of visual stimuli (neutral, anxiogenic, mutilation)  Second and third finger of the non-dominant hand  Biopac | Patients had greater skin conductance responses during all stimuli | 2 |
| Wise, McFarlane, Clark, and Battersby (2009) | N=50 (35 female, 15 male)  Mean age: 35.8±13.4 years  Inclusion: panic disorder according to the DSM-IV  Exclusion: substance abuse, bipolar disorder, history of neurological disorders, brain injury, serious medical problem, benzodiazepine intake | N=98 (69 female, 29 male)  Mean age: 35.6±13.1 years | At baseline, during an auditory oddball paradigm  Fingers of the non-dominant hand | No difference in skin conductance responses between patients and controls | 4 |
| Burkhardt et al. (2010) | N=25  Mean age: 39.7±10.7 years  Inclusion: panic disorder according to the DSM-IV  Exclusion: psychosis, drug dependence or abuse, history of bipolar disorder, mental disability, somatic diseases that might affect the physiological measurements | N=20  Mean age: 38.4±10.9 years | During 15 min of quiet sitting  Middle phalanges of the left index and middle fingers | No differences in skin conductance levels between patients and controls | 4 |
| Doberenz, Roth, Wollburg, Breuninger, and Kim (2010) | N=22 (14 female, 8 male)  Mean age: 42.7±15.0 years  Inclusion: panic disorder according to the DSM-IV  Exclusion: cognitive impairment, substance abuse or dependence in the past year, history of psychosis, intake of drugs with substantial anticholinergic effects | N=29 (19 female, 10 male)  Mean age: 42.1±12.3 years | Ambulatory monitoring for a 24-hour period, including four relaxation tests (walking at normal pace followed by quiet sitting)  Middle or lower phalanges of the index and middle finger of the non-dominant hand  BioLog | Patients had higher day- and night-time skin conductance levels | 5 |
| Favaron et al. (2010) | N=29 (17 female, 12 male)  Mean age: 34.7±9.7 years  Inclusion: panic disorder with agoraphobia according to the DSM-IV  Exclusion: mental disorders, major physical diseases, psychotropic medication in the 2 weeks prior to testing | N=29 (17 female, 12 male)  Mean age: 32.3±8.4 years | At baseline, during neutral startling sounds (100 dB)  Second and third finger of the non-dominant hand  Biopac | No differences in skin conductance levels and skin conductance responses between patients and controls | 2 |
| Freire, De Carvalho, Joffily, Zin, and Nardi (2010) | N=10 (6 female, 4 male)  Mean age: 39.2±9.6 years  Inclusion: panic disorder with agoraphobia according to the DSM-IV, at least two panic attacks in the month prior to testing  Exclusion: mental disorders (except major depression), neurological, cardiologic or respiratory diseases | N=9 (6 female, 3 male)  Mean age: 37.7±10.9 years | During an animation film about an agoraphobia-related situation  Biopac | Patients had higher skin conductance levels and greater skin conductance responses | 1 |
| McTeague, Lang, Laplante, and Bradley (2011) | N=112 (sex distribution not stated)  Mean age: 29.1±10.80years (panic disorder without agoraphobia), 36.91±14.68 years (panic disorder with moderate agoraphobia), 35.22±12.46 years (panic disorder with severe agoraphobia)  Inclusion: panic disorder according to the DSM-IV | N=76 (50 female, 26 male)  Mean age: 31.8±11.6 | At baseline, during an imagery task (neutral, panic attack, survival threat, personal threat)  Hypothenar eminence of the non-dominant hand  Coulbourn | No differences in skin conductance levels between patients and controls | 2 |
| Wise, McFarlane, Clark, and Battersby (2011) | N=52 (37 female, 15 male)  Mean age: 35.3±13.4 years  Inclusion: panic disorder according to the DSM-IV  Exclusion: substance abuse, history of neurological disorders, brain injury, serious medical problem, benzodiazepine intake within 2 weeks | N=104 (74 female, 30 male)  Mean age: 35±13.1 years | Resting eyes open vs. closed  Fingers of the non-dominant hand | Patients had fewer non-specific skin conductance fluctuations during both conditions and lower decrements in skin conductance levels when having their eyes open | 4 |
| Otto et al. (2014) | N=24 (11 female, 13 male)  Mean age: 32.5±7.9 years  Inclusion: panic disorder according to the DSM-IV  Exclusion: pregnancy, substance use disorder, past or present psychosis, suicidality or homicidality, serious medical conditions, use of beta-blockers | N=102 (47 female, 55 male)  Mean age: 29.6±10.3 years | During habituation, fear acquisition, and extinction  Hypothenar eminence of the non-dominant hand  Coulbourn | No differences in skin conductance responses between patients and controls | 2 |

DSM=Diagnostic and Statistical Manual of Mental Disorders

**Supplement 6** Characteristics of included studies investigating skin conductance in patients with generalised anxiety disorder

| Study | Patients | Controls | Measurements | Main results | Risk of bias (0-8) |
| --- | --- | --- | --- | --- | --- |
| Orr and Pitman (1987) | N=20 (all male)  Mean age: 45.2 years  Inclusion: generalised anxiety disorder according to the DSM-III  Exclusion: organic mental disorder, substance dependence, psychotic disorder, major affective disorder, posttraumatic stress disorder, medication intake | N=20 (all male)  Mean age: 45.5 years | At baseline, during acoustic stimulation (86 dB)  Palm of the left hand  Coulbourn | Patients had more non-specific skin conductance fluctuations at baseline | 3 |
| Hoehn-Saric, McLeod, and Zimmerli (1989) | N=20 (all female)  Mean age: 36.1±7.6 years  Inclusion: generalised anxiety disorder according to the DSM-III-R, a score ≥ 38 on the trait version of the State Trait Anxiety Inventory score, Hamilton Anxiety Rating Scale score ≥ 18  Exclusion: history of major mental illness, history of panic attacks, medication affecting the central or autonomic nervous system 2 weeks prior to testing | N=20 (all female)  Mean age: 34.6±7.9 years | At baseline, during a divided attention task and during a risk-taking task  Middle phalanges of the index and middle fingers of the non-dominant hand  J&J Company | Patients had lower skin conductance levels during the risk-taking task | 3 |
| Bruce et al. (1992) | N=12 (3 female, 9 male)  Mean age: 36 years  Inclusion: generalised anxiety disorder according to the DSM-III, ≥18 on Hamilton Anxiety Rating Scale and <14 on Hamilton Rating Scale for Depression  Exclusion: pregnancy, substance abuse, abnormal laboratory test results, psychotropic drugs (other than anxiolytics) | N=12 (6 female, 6 male)  Mean age: 34 years | During a caffeine challenge | Patients had higher skin conductance levels | 3 |
| Roth et al. (1998b) | N=17  Mean age: 39.4±10.8 years  Inclusion: generalised anxiety disorder according to the DSM-III-R  Exclusion: depressive episode, respiratory diseases, psychoactive or cardiovascularly active medication in the 2 weeks before testing | N=22  Mean age: 44.8±16.4 years | At baseline, during breath holding  Vitaport | No differences in skin conductance levels between patients and controls | 4 |
| Wilhelm, Trabert, et al. (2001a) and Wilhelm, Trabert, et al. (2001b) | N=15 (9 female, 6 male)  Mean age: 37.3 ± 9.0 years  Inclusion: generalised anxiety disorder according to the DSM-III  Exclusion: major depressive episode, epileptic, respiratory, or cardiovascular disease, psychoactive or cardiovascularly active medication in the 2 weeks prior to testing | N=19 (12 female, 7 male)  Mean age: 43.7±16.1 years | During 30 min of quiet sitting  Digits 3 and 4 of the left hand  Vitaport | No differences in skin conductance levels and non-specific skin conductance fluctuations between patients and controls | 3 |
| Hoehn-Saric et al. (2004) | N=40 generalised anxiety disorder (29 female, 11 male)  Mean age: 39.6±9.2  N=26 panic disorder (22 female, 4 male)  Mean age: 36.1±8.4  Inclusion: generalised anxiety disorder or panic disorder according to the DSM-IV, a score ≥38 on the trait version of the State Trait Anxiety Inventory and ≥18 on the Hamilton Rating Scale for Anxiety  Exclusion: mental disorders (except mild phobias), medication affecting the central and autonomic nervous system in the 2 weeks prior to testing | n=24 (17 female, 7 male)  Mean age: 31.6±7.7 | Ambulatory monitoring between 9 am and 5 pm  2 fingers of the non-dominant hand | No differences in skin conductance levels between patients and controls when stress was reported | 5 |
| Andor, Gerlach, and Rist (2008) | N=33 (24 female, 9 male)  Inclusion: generalised anxiety disorder according to the DSM-IV  Mean age: 37.2±11.4 years | N=34 controls (24 female, 10 male)  Mean age: 37.4±11.3 years | During a signal detection task  Palm of the hand  Vitaport | No differences in skin conductance levels and non-specific skin conductance fluctuations between patients and controls | 3 |
| Upatel and Gerlach (2008) | N=32 (19 female, 13 male)  Mean age: 40.5±11.9 years  Inclusion: generalised anxiety disorder according to the DSM-IV | N=31 (19 female, 13 male)  Mean age: 41.5±12.7 years | At baseline, during a film presentation, and during thought sampling  Palm of the hand  Vitaport | No differences in skin conductance levels and non-specific skin conductance fluctuations between patients and controls | 3 |
| Fisher, Granger, and Newman (2010) | N=45 (35 female, 10 male)  Mean age: 19.9±1 years  Inclusion: generalised anxiety disorder according to the DSM-IV | N=62 (40 female, 22 male)  Mean age: 19.5±0.8 years | At baseline, during a negatively valenced film clip  Distal phalanges of the first and second finger of the non-dominant hand  Biopac | No differences in skin conductance levels and non-specific skin conductance fluctuations between patients and controls | 2 |
| Yassa, Hazlett, Stark, and Hoehn-Saric (2012) | N=15 (12 female, 3 male)  Mean age: 34.7±9.5 years  Inclusion: generalised anxiety disorder according to the DSM-IV  Exclusion: pathologies, medication intake | N=15 (9 female, 6 male)  Mean age: 32.5±8.7 years | During a gambling task  Palm of the non-dominant hand  Contact Precision Instruments | Patients started with lower skin conductance responses, which remained elevated at the end of the task | 5 |

DSM=Diagnostic and Statistical Manual of Mental Disorders

**Supplement 7** Characteristics of included studies investigating body temperature in patients with panic disorder

| Study | Patients | Controls | Measurement | Main results | Risk of bias (0-8) |
| --- | --- | --- | --- | --- | --- |
| Freedman et al. (1984) | N=8 (4 female, 4 male)  Inclusion: panic disorder according to the DSM-III | N=9 (4 female, 5 male) | During infusion (placebo, sodium lactate, isoproterenol)  Distal end of the middle finger of each hand  Yellow Springs | Patients had lower finger temperature levels before and during all three infusions | 1 |
| Freedman, Ianni, Ettedgui, and Puthezhath (1985) | N=12 (9 female, 3 male)  Mean age: 35±10.5 years  Inclusion: panic disorder according to the DSM-III  Exclusion: medication | N=11 (10 female, 1 male)  Mean age: 36.6±13.4 years | Ambulatory monitoring between 10 am and 10 pm for two consecutive days  Distal end of the middle finger of one hand | No differences in finger temperature between patients and controls | 3 |
| Cameron, Lee, Kotun, and McPhee (1986) | N=11 (9 female, 2 male)  n=8 panic disorder  n=3 agoraphobia with panic attacks  Mean age: 35 years  Inclusion: panic disorder according to the DSM-III | N=6 controls (4 female, 2 male)  Mean age: 32 years | Ambulatory monitoring at five times of the day (7 am, 11 am, 3 pm, 7 pm, 11 pm) for one week  Oral temperature | No differences in oral temperature between patients and controls | 1 |
| Lesch (1991) and Lesch et al. (1992) | N=14 (9 women, 5 men)  Mean age: 30.6+6.5 years  Inclusion: panic disorder according to the DSM-III-R  Exclusion: drugs within 3 weeks | N=14 (9 women, 5 men)  Mean age: 33.4+12.3 years | At baseline, during ipsapirone and placebo administration  Oral temperature | Patients had an attenuated hypothermic response to ipsapirone | 4 |
| Borden et al. (1993) | N=19 (10 female, 9 male)  Mean age: 43.3 years (range: 31-64)  Inclusion: panic disorder according to the DSM-III  Exclusion: mental disorders | N=20 (10 female, 10 male)  Mean age: 20.3±4.11 years | At baseline, during relaxation, and during a mental arithmetic task  Dorsal surface of the fourth finger of the non-dominant hand  J&J Company | No differences in finger temperature between patients and controls | 3 |
| Craske and Freed (1995) | N=18 (12 female, 6 male)  Mean age: 35.3±10.6 years  Inclusion: panic disorder according to the DSM-III-R  Exclusion: organic brain disorder, drug abuse-dependence within the last 6 months, any history of psychosis, suicidal ideation, mental retardation, cardiovascular, neurological, or other medical conditions placing individuals at risk during arousal, recent changes in medication | N=18 (12 female, 6 male)  Mean age: 28.9±9.1 years | During relaxation and during audio feedback reflecting heightened expected vs. unexpected arousal  Tip of the little finger of the non-dominant hand  Coulbourn | Patients had higher finger temperature in the unexpected arousal condition | 4 |
| Roth et al. (1998a) | N=14 (8 female, 6 male)  Mean age: 41.6±10.2 years  Inclusion: panic disorder according to the DSM-IV  Exclusion: psychoactive or cardiovascularly active medication in the 2 weeks prior to testing | N=15 (9 female, 6 male)  Mean age: 40.3±12.6 years | During talking, relaxation, and talking  Distal phalanx of the left fifth digit  Vitaport | Patients had more pronounced increases in finger temperature during the relaxation task | 3 |
| Broocks et al. (2000) | N=40 (20 female, 20 male)  Mean age: 32.1±8.7 years  Inclusion: panic disorder according to the DSM-IV and ICD-10  Exclusion: pregnancy, lactation, body weight below 80% of ideal body weight, drug dependency, psychotic symptoms, bipolar disorder, anorexia or bulimia nervosa, significant medical illness, psychotropic medication within 3 weeks | N=12 (6 female, 6 male)  Mean age: 33.45±5.4 years | During ipsapirone and placebo administration  Oral temperature | No differences in the hypothermic response to ipsapirone between patients and controls | 5 |
| Bystritsky, Maidenberg, et al. (2000) | N=48 (28 female, 20 male)  Mean age: 35.8±10.6 years  Inclusion: panic disorder according to the DSM-III-R  Exclusion: pregnancy, organic brain damage, alcohol or drug abuse, psychosis, current major depressive episode, personality disorder,  significant health impairments, use of psychotropic drugs | N=24 (13 female, 11 male)  Mean age: 34.5±10.7 years | At baseline, during various tasks (relaxation, orthostatic challenge, hyperventilation, Valsalva, acoustic stimulation, mental arithmetic, handgrip, knee-bends, imagery)  Thumb of the right hand  J&J Company | No differences in finger temperature between patients and controls | 5 |
| Craske et al. (2001) | N=90  Mean age: total sample 34.7±9.5 years  Inclusion: panic disorder according to the DSM-IV  Exclusion: pregnancy, severe obesity, hearing impairment, substance abuse, psychotic disorder, bipolar disorder, dissociative states, history of post-traumatic stress disorder, neurological, cardiovascular, respiratory or renal diseases, pheochromocytoma, hypo- or hyperthyroidism, amphetamine intoxication, psychoactive medication | N=16  Mean age: total sample 34.7±9.5 years | At baseline, during a cardiac tracking, a meditative relaxation task, and a hyperventilation task  Tip of the little finger of the non-dominant hand  Coulbourn | Patients had lower finger temperature levels during the relaxation task | 4 |
| Michael et al. (2007) | N=39  Mean age: 40.3±10.6 years  Inclusion: panic disorder according to the DSM-IV  Exclusion: alcohol or other substance abuse in the past year, psychotic disorders, bipolar disorder, history of conditions that might affect the physiological systems under study, use of medication with strong autonomic effects | N=33  Mean age: 42.6±8.96 years | At baseline  Biopac | No differences in skin temperature between patients and controls | 4 |
| Todder, Fox, and Baune (2009) | N=12 (all female)  Mean age: 32.8±11 years  Inclusion: panic disorder according to the DSM-IV  Exclusion: mental disorders, suicidal ideation, significant organic disorders, endocrine disorders, treatment for panic disorder | N=12 (all female)  Mean age: 36.3±10.0 years | Ambulatory measurement for five weeks  Wrist  Actiwatch | No differences in wrist temperature between patients and controls | 3 |

DSM=Diagnostic and Statistical Manual of Mental Disorders

**References**

Ahrens, L. M., Pauli, P., Reif, A., Muhlberger, A., Langs, G., Aalderink, T., & Wieser, M. J. (2016). Fear conditioning and stimulus generalization in patients with social anxiety disorder. *J Anxiety Disord, 44*, 36-46.

Albus, M., Braune, S., Hohn, T., & Scheibe, G. (1988). Do anxiety patients with or without frequent panic attacks differ in their response to stress. *Stress Medicine, 4*, 189-194.

Alpers, G. W., Wilhelm, F. H., & Roth, W. T. (2005). Psychophysiological assessment during exposure in driving phobic patients. *J Abnorm Psychol, 114*, 126-139.

Andor, T., Gerlach, A. L., & Rist, F. (2008). Superior perception of phasic physiological arousal and the detrimental consequences of the conviction to be aroused on worrying and metacognitions in GAD. *J Abnorm Psychol, 117*, 193-205.

Argyle, N. (1991). Skin conductance levels in panic disorder and depression. *J Nerv Ment Dis, 179*, 563-566.

Beck, J. G., Stanley, M. A., Averill, P. M., Baldwin, L. E., & Deagle, E. A., 3rd. (1992). Attention and memory for threat in panic disorder. *Behav Res Ther, 30*, 619-629.

Blechert, J., Michael, T., Grossman, P., Lajtman, M., & Wilhelm, F. H. (2007). Autonomic and respiratory characteristics of posttraumatic stress disorder and panic disorder. *Psychosomatic medicine, 69*, 935-943.

Borden, J. W., Lowenbraun, P. B., Wolff, P. L., & Jones, A. (1993). Self-Focused Attention in Panic Disorder. *Cognitive Therapy and Research, 17*, 413-425.

Braune, S., Albus, M., Frohler, M., Hohn, T., & Scheibe, G. (1994). Psychophysiological and biochemical changes in patients with panic attacks in a defined situational arousal. *Eur Arch Psychiatry Clin Neurosci, 244*, 86-92.

Broocks, A., Bandelow, B., George, A., Jestrabeck, C., Opitz, M., Bartmann, U., . . . Hajak, G. (2000). Increased psychological responses and divergent neuroendocrine responses to m-CPP and ipsapirone in patients with panic disorder. *Int Clin Psychopharmacol, 15*, 153-161.

Bruce, M., Scott, N., Shine, P., & Lader, M. (1992). Anxiogenic effects of caffeine in patients with anxiety disorders. *Arch Gen Psychiatry, 49*, 867-869.

Burkhardt, S. C., Wilhelm, F. H., Meuret, A. E., Blechert, J., & Roth, W. T. (2010). Temporal stability and coherence of anxiety, dyspnea, and physiological variables in panic disorder. *Biological psychology, 85*, 226-232.

Bystritsky, A., Craske, M., Maidenberg, E., Vapnik, T., & Shapiro, D. (2000). Autonomic reactivity of panic patients during a CO2 inhalation procedure. *Depress Anxiety, 11*, 15-26.

Bystritsky, A., Maidenberg, E., Craske, M. G., Vapnik, T., & Shapiro, D. (2000). Laboratory psychophysiological assessment and imagery exposure in panic disorder patients. *Depress Anxiety, 12*, 102-108.

Cameron, O. G., Lee, M. A., Kotun, J., & McPhee, K. M. (1986). Circadian symptom fluctuations in people with anxiety disorders. *J Affect Disord, 11*, 213-218.

Craske, M. G., & Freed, S. (1995). Expectations about arousal and nocturnal panic. *J Abnorm Psychol, 104*, 567-575.

Craske, M. G., Lang, A. J., Tsao, J. C., Mystkowski, J. L., & Rowe, M. K. (2001). Reactivity to interoceptive cues in nocturnal panic. *J Behav Ther Exp Psychiatry, 32*, 173-190.

Cuthbert, B. N., Lang, P. J., Strauss, C., Drobes, D., Patrick, C. J., & Bradley, M. M. (2003). The psychophysiology of anxiety disorder: fear memory imagery. *Psychophysiology, 40*, 407-422.

Del-Ben, C. M., Vilela, J. A. A., Hetem, L. A. B., Guimaraes, F. S., Graeff, F. G., & Zuardi, A. W. (2001). Do panic patients process unconditioned fear vs. conditioned anxiety differently than normal subjects? *Psychiatry Res, 104*, 227-237.

Diemer, J., Lohkamp, N., Muhlberger, A., & Zwanzger, P. (2016). Fear and physiological arousal during a virtual height challenge--effects in patients with acrophobia and healthy controls. *J Anxiety Disord, 37*, 30-39.

Doberenz, S., Roth, W. T., Wollburg, E., Breuninger, C., & Kim, S. (2010). Twenty-four hour skin conductance in panic disorder. *J Psychiat Res, 44*, 1137-1147.

Edelmann, R. J., & Baker, S. R. (2002). Self-reported and actual physiological responses in social phobia. *Br J Clin Psychol, 41*, 1-14.

Favaron, E., Bellodi, L., Biffi, S., Vanni, G., Zorzi, C., Liperi, L., & Perna, G. (2010). Acoustic startle response in panic disorder. *Psychiatry Res, 176*, 254-256.

Fisher, A. J., Granger, D. A., & Newman, M. G. (2010). Sympathetic arousal moderates self-reported physiological arousal symptoms at baseline and physiological flexibility in response to a stressor in generalized anxiety disorder. *Biological psychology, 83*, 191-200.

Freedman, R. R., Ianni, P., Ettedgui, E., Pohl, R., & Rainey, J. M. (1984). Psychophysiological factors in panic disorder. *Psychopathology, 17 Suppl 1*, 66-73.

Freedman, R. R., Ianni, P., Ettedgui, E., & Puthezhath, N. (1985). Ambulatory Monitoring of Panic Disorder. *Arch Gen Psychiatry, 42*, 244-248.

Freire, R. C., De Carvalho, M. R., Joffily, M., Zin, W. A., & Nardi, A. E. (2010). Anxiogenic properties of a computer simulation for panic disorder with agoraphobia. *J Affect Disord, 125*, 301-306.

Gerlach, A. L., Wilhelm, F. H., Gruber, K., & Roth, W. T. (2001). Blushing and physiological arousability in social phobia. *J Abnorm Psychol, 110*, 247-258.

Gerlach, A. L., Wilhelm, F. H., & Roth, W. T. (2003). Embarrassment and social phobia: the role of parasympathetic activation. *J Anxiety Disord, 17*, 197-210.

Hermann, C., Ziegler, S., Birbaumer, N., & Flor, H. (2002). Psychophysiological and subjective indicators of aversive pavlovian conditioning in generalized social phobia. *Biological psychiatry, 52*, 328-337.

Hoehn-Saric, R., McLeod, D. R., Funderburk, F., & Kowalski, P. (2004). Somatic symptoms and physiologic responses in generalized anxiety disorder and panic disorder: an ambulatory monitor study. *Arch Gen Psychiatry, 61*, 913-921.

Hoehn-Saric, R., McLeod, D. R., & Zimmerli, W. D. (1989). Somatic manifestations in women with generalized anxiety disorder. Psychophysiological responses to psychological stress. *Arch Gen Psychiatry, 46*, 1113-1119.

Hoehn-Saric, R., McLeod, D. R., & Zimmerli, W. D. (1991). Psychophysiological response patterns in panic disorder. *Acta Psychiatr Scand, 83*, 4-11.

Hoehn, T., Braune, S., Scheibe, G., & Albus, M. (1997). Physiological, biochemical and subjective parameters in anxiety patients with panic disorder during stress exposure as compared with healthy controls. *Eur Arch Psychiatry Clin Neurosci, 247*, 264-274.

Knopf, K., & Possel, P. (2009). Individual response differences in spider phobia: comparing phobic and non-phobic women of different reactivity levels. *Anxiety Stress Coping, 22*, 39-55.

Lesch, K. P. (1991). 5-HT1A receptor responsivity in anxiety disorders and depression. *Prog Neuropsychopharmacol Biol Psychiatry, 15*, 723-733.

Lesch, K. P., Wiesmann, M., Hoh, A., Muller, T., Disselkamp-Tietze, J., Osterheider, M., & Schulte, H. M. (1992). 5-HT1A receptor-effector system responsivity in panic disorder. *Psychopharmacology (Berl), 106*, 111-117.

Li, S., & Graham, B. M. (2016). Estradiol is associated with altered cognitive and physiological responses during fear conditioning and extinction in healthy and spider phobic women. *Behav Neurosci, 130*, 614-623.

Lopes, F. L., Azevedo, T. M., Imbiriba, L. A., Freire, R. C., Valenca, A. M., Caldirola, D., . . . Nardi, A. E. (2009). Freezing reaction in panic disorder patients associated with anticipatory anxiety. *Depress Anxiety, 26*, 917-921.

McTeague, L. M., Lang, P. J., Laplante, M. C., & Bradley, M. M. (2011). Aversive imagery in panic disorder: agoraphobia severity, comorbidity, and defensive physiology. *Biological psychiatry, 70*, 415-424.

McTeague, L. M., Lang, P. J., Laplante, M. C., Cuthbert, B. N., Strauss, C. C., & Bradley, M. M. (2009). Fearful imagery in social phobia: generalization, comorbidity, and physiological reactivity. *Biological psychiatry, 65*, 374-382.

McTeague, L. M., Lang, P. J., Wangelin, B. C., Laplante, M. C., & Bradley, M. M. (2012). Defensive mobilization in specific phobia: fear specificity, negative affectivity, and diagnostic prominence. *Biological psychiatry, 72*, 8-18.

Michael, T., Blechert, J., Vriends, N., Margraf, J., & Wilhelm, F. H. (2007). Fear conditioning in panic disorder: Enhanced resistance to extinction. *J Abnorm Psychol, 116*, 612-617.

Moscovitch, D. A., Suvak, M. K., & Hofmann, S. G. (2010). Emotional response patterns during social threat in individuals with generalized social anxiety disorder and non-anxious controls. *J Anxiety Disord, 24*, 785-791.

Muehlberger, A., Petrusek, S., Herrmann, M. J., & Pauli, P. (2005). Biocyber psychology: subjective and physiological reactions in flight phobics and normal subjects during flight simulations. *Zeitschrift Fur Klinische Psychologie Und Psychotherapie, 34*, 133-143.

Notzon, S., Deppermann, S., Fallgatter, A., Diemer, J., Kroczek, A., Domschke, K., . . . Ehlis, A. C. (2015). Psychophysiological effects of an iTBS modulated virtual reality challenge including participants with spider phobia. *Biological psychology, 112*, 66-76.

Orr, S. P., & Pitman, R. K. (1987). Electrodermal psychophysiology of anxiety disorder: orienting response and spontaneous fluctuations. *Biological psychiatry, 22*, 653-656.

Otto, M. W., Moshier, S. J., Kinner, D. G., Simon, N. M., Pollack, M. H., & Orr, S. P. (2014). De novo fear conditioning across diagnostic groups in the affective disorders: evidence for learning impairments. *Behavior Therapy, 45*, 619-629.

Owens, M. E., & Beidel, D. C. (2015). Can virtual reality effectively elicit distress associated with social anxiety disorder? *Journal of Psychopathology and Behavioral Assessment, 37*, 296-305.

Parente, A. C., Garcia-Leal, C., Del-Ben, C. M., Guimaraes, F. S., & Graeff, F. G. (2005). Subjective and neurovegetative changes in healthy volunteers and panic patients performing simulated public speaking. *Eur Neuropsychopharmacol, 15*, 663-671.

Passchier, J., Verheij, R., Tulen, J. H., Timmerman, L., & Pepplinkhuizen, L. (1992). Positive associations between anticipatory anxiety and needle pain for subjective but not for physiological measures of anxiety. *Psychol Rep, 70*, 1059-1062.

Peperkorn, H. M., Alpers, G. W., & Muhlberger, A. (2014). Triggers of fear: perceptual cues versus conceptual information in spider phobia. *J Clin Psychol, 70*, 704-714.

Ritz, T., Wilhelm, F. H., Meuret, A. E., Gerlach, A. L., & Roth, W. T. (2011). Airway response to emotion- and disease-specific films in asthma, blood phobia, and health. *Psychophysiology, 48*, 121-135.

Roth, W. T., Ehlers, A., Taylor, C. B., Margraf, J., & Agras, W. S. (1990). Skin conductance habituation in panic disorder patients. *Biological psychiatry, 27*, 1231-1243.

Roth, W. T., Margraf, J., Ehlers, A., Taylor, C. B., Maddock, R. J., Davies, S., & Agras, W. S. (1992). Stress test reactivity in panic disorder. *Arch Gen Psychiatry, 49*, 301-310.

Roth, W. T., Telch, M. J., Taylor, C. B., Sachitano, J. A., Gallen, C. C., Kopell, M. L., . . . Pfefferbaum, A. (1986). Autonomic characteristics of agoraphobia with panic attacks. *Biological psychiatry, 21*, 1133-1154.

Roth, W. T., Wilhelm, F. H., & Trabert, W. (1998a). Autonomic instability during relaxation in panic disorder. *Psychiatry Res, 80*, 155-164.

Roth, W. T., Wilhelm, F. H., & Trabert, W. (1998b). Voluntary breath holding in panic and generalized anxiety disorders. *Psychosomatic medicine, 60*, 671-679.

Sansen, L. M., Iffland, B., & Neuner, F. (2015). The trauma of peer victimization: psychophysiological and emotional characteristics of memory imagery in subjects with social anxiety disorder. *Psychophysiology, 52*, 107-116.

Sartory, G., Heinen, R., Wannemüller, A., Lohrmann, T., & Jöhren, P. (2009). Die modulierte Schreckreaktion bei Zahnbehandlungsphobie. *Zeitschrift Fur Klinische Psychologie Und Psychotherapie, 38*, 213-222.

Shiban, Y., Peperkorn, H., Alpers, G. W., Pauli, P., & Muhlberger, A. (2016). Influence of perceptual cues and conceptual information on the activation and reduction of claustrophobic fear. *J Behav Ther Exp Psychiatry, 51*, 19-26.

Sigmon, S. T., Dorhofer, D. M., Rohan, K. J., Hotovy, L. A., Boulard, N. E., & Fink, C. M. (2000). Psychophysiological, somatic, and affective changes across the menstrual cycle in women with panic disorder. *J Consult Clin Psychol, 68*, 425-431.

Strauman, T. J. (1989). Self-discrepancies in clinical depression and social phobia: cognitive structures that underlie emotional disorders? *J Abnorm Psychol, 98*, 14-22.

Todder, D., Fox, M., & Baune, B. T. (2009). Body temperature in patients with panic disorder treated with escitalopram. *Clin Auton Res, 19*, 255-258.

Upatel, T., & Gerlach, A. L. (2008). Appraisal of activating thoughts in generalized anxiety disorder. *Journal of Behavior Therapy and Experimental Psychiatry, 39*, 234-249.

Voncken, M. J., & Bogels, S. M. (2009). Physiological blushing in social anxiety disorder patients with and without blushing complaints: Two subtypes? *Biological psychology, 81*, 86-94.

Wessel, I., & Merckelbach, H. (1998). Memory for threat-relevant and threat-irrelevant cues in spider phobics. *Cognition & Emotion, 12*, 93-104.

Whittal, M. L., & Goetsch, V. L. (1995). Physiological, subjective and behavioral responses to hyperventilation in clinical and infrequent panic. *Behav Res Ther, 33*, 415-422.

Wikstrom, J., Lundh, L. G., Westerlund, J., & Hogman, L. (2004). Preattentive bias for snake words in snake phobia? *Behav Res Ther, 42*, 949-970.

Wilhelm, F. H., Gerlach, A. L., & Roth, W. T. (2001). Slow recovery from voluntary hyperventilation in panic disorder. *Psychosomatic medicine, 63*, 638-649.

Wilhelm, F. H., & Roth, W. T. (1998). Taking the laboratory to the skies: ambulatory assessment of self-report, autonomic, and respiratory responses in flying phobia. *Psychophysiology, 35*, 596-606.

Wilhelm, F. H., Trabert, W., & Roth, W. T. (2001a). Characteristics of sighing in panic disorder. *Biological psychiatry, 49*, 606-614.

Wilhelm, F. H., Trabert, W., & Roth, W. T. (2001b). Physiologic instability in panic disorder and generalized anxiety disorder. *Biological psychiatry, 49*, 596-605.

Wise, V., McFarlane, A. C., Clark, C. R., & Battersby, M. (2009). Event-related potential and autonomic signs of maladaptive information processing during an auditory oddball task in panic disorder. *International Journal of Psychophysiology, 74*, 34-44.

Wise, V., McFarlane, A. C., Clark, C. R., & Battersby, M. (2011). An integrative assessment of brain and body function 'at rest' in panic disorder: a combined quantitative EEG/autonomic function study. *Int J Psychophysiol, 79*, 155-165.

Wollburg, E., Meuret, A. E., Conrad, A., Roth, W. T., & Kim, S. (2008). Psychophysiological reactions to two levels of voluntary hyperventilation in panic disorder. *J Anxiety Disord, 22*, 886-898.

Yassa, M. A., Hazlett, R. L., Stark, C. E., & Hoehn-Saric, R. (2012). Functional MRI of the amygdala and bed nucleus of the stria terminalis during conditions of uncertainty in generalized anxiety disorder. *J Psychiat Res, 46*, 1045-1052.
